# Characterization of rare trifucosylated human milk oligosaccharides by cryogenic infrared ion spectroscopy (CIRIS)

**DOI:** 10.1007/s00216-025-06114-5

**Published:** 2025-09-26

**Authors:** Ali H. Abikhodr, Stephan Warnke, Ahmed Ben Faleh, Thomas R. Rizzo, Sibel Goeraler, John Gonsalves, Bernd Stahl, Marko Mank

**Affiliations:** 1 Isospec Analytics SA, CH-1020 Renens, Switzerland; 2 École Polytechnique Fédérale de Lausanne, EPFL SB ISIC, Station 6, CH-1015 Lausanne, Switzerland; 3 Danone Research and Innovation, 3584 CT Utrecht, The Netherlands; 4 Department of Chemical Biology and Drug Discovery, Utrecht Institute for Pharmaceutical Sciences, Utrecht University, 3584 CG Utrecht, The Netherlands

**Keywords:** Cryogenic IR spectroscopy, Glycan analysis, Human milk oligosaccharides, TF-LNT, Structural analysis of glycomics, Mass spectrometry, Human milk oligosaccharides

## Abstract

While approximately 200 human milk oligosaccharide structures (HMOs) have been completely characterized, the precise isomeric structures of even more HMOs remain ambiguous. To address this analytical challenge, we employ tandem mass spectrometry (i.e., MS/MS) coupled with cryogenic infrared ion spectroscopy (CIRIS) to characterize and identify novel fucosylated HMOs isolated from pooled human milk. In this approach, the precise structure of isomeric precursor molecules purified by liquid chromatography (LC) is identified by IR fingerprinting their fragments. Using this technique, we have identified four novel isomers of trifucosylated oligosaccharides (i.e., trifucosyl-lacto-N-heptaoses and trifucosyl-lacto-N-neoheptaoses), some of which could not have been produced by previously known biosynthetic pathways for HMO synthesis in the mammary gland.

**Graphical Abstract:**

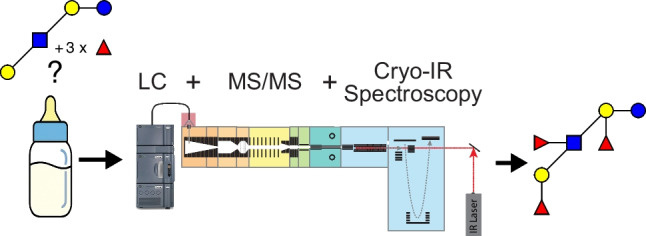

**Supplementary Information:**

The online version contains supplementary material available at 10.1007/s00216-025-06114-5.

## Introduction

Human milk oligosaccharides (HMOs) comprise the third most abundant fraction of constituents in human milk after lactose and fat [1–3]. These complex non-digestible glycans play an important role in supporting an infant’s gut microbiota and immune system development [4–14]. For this reason, HMOs are being increasingly incorporated into infant formula to mimic the functional advantages of human milk.

Composed of five primary monosaccharides—glucose, galactose, fucose, N-acetylglucosamine, and sialic acid—HMOs are built upon a lactose core, which can be extended with additional monosaccharide units in linear or branched chains. Further modification through fucosylation or sialylation adds to their complexity. While the theoretical number of possible HMO isomers is immense [15], the actual diversity in biological systems is limited by the human glycosyltransferases that synthesize them. For example, the β−1,3-N-acetylglucosaminyltransferase family of enzymes (B3GNTs) extends the lactose core by catalyzing the addition of a GlcNAc unit to the terminal galactose residue via a β−1,3 linkage [16], while the fucosyltransferases FUT2 and FUT3 add fucose via α−1,2 or α−1,3/4 linkages, respectively. The substrate specificity of these enzymes severely reduces the number of possible isomers found in human milk and accounts for their characteristic structural patterns [17, 18].

Determining the precise structure of individual HMO isomers presents a formidable analytical challenge, however. Tandem mass spectrometry (MS/MS) coupled with liquid chromatography (LC) has become the standard method for oligosaccharide analysis, largely because of the high sensitivity of MS and the discriminatory power of LC. However, in many cases this approach cannot distinguish the subtly different isomers that one finds in human milk. Our approach to overcoming this problem is to add an entirely new dimension to biomolecular identification by measuring a cryogenic infrared spectrum inside a mass spectrometer [19–23]. This approach, which we call cryogenic infrared ion spectroscopy (CIRIS), provides a robust molecular fingerprint consisting of a set of distinct peaks that are unique to a given isomeric species. This allows us to distinguish and identify the subtlest differences in oligosaccharide isomers. Moreover, this approach maintains the high level of sensitivity afforded by MS. We have used this technique to create an ever-growing database of HMO infrared fingerprints with which to distinguish and identify isomeric species. For cases in which we encounter an isomeric structure that is not already contained in our database, we have developed a fragmentation-based scheme to identify isomeric fragment ions that can then be pieced back together to determine the precise isomeric structure of the precursor molecular ion [20, 23–25].

In this present work, we apply this approach to identify HMO isomers derived from a pooled human milk sample. We report here four novel, triply-fucosylated HMO species, two of which cannot be explained by known biosynthetic pathways in the mammary gland.

## Experimental methods

### Preparation of defined HMO fractions

A total carbohydrate mineral fraction was isolated from pooled mature human milk as essentially described by Kobata et al. [26] and Finke et al. [27]. Two grams of this total carbohydrate mineral fraction was then dissolved in 5 ml MilliQ water and subsequently subjected to preparative size exclusion chromatography (SEC) in order to deplete lactose and yield further defined HMO fractions. The SEC stationary phase consisted of TOYOPEARL® HW-40S (Tosoh Bioscience GmbH) gel in two serially coupled KRONLAB ECO50 columns of 1000 mm and 1200 mm in length. The columns have an inner diameter of 50 mm, and the frits which prevented the gel from leaking out of the columns have a pore size of 10–16 µm. Further modules of the preparative SEC system were from Agilent: a 1260 Infinity II Isocratic Pump, a 1260 Infinity II Refractive Index detector, and a 1290 Infinity II Preparative Open-Bed Fraction Collector.

The defined HMO fractions comprised a total lactose-depleted HMO fraction, a total acidic HMO fraction, and a neutral HMO fraction ranging in degree of polymerization between 6 and 9 (i.e., DP6 to DP9). These fractions were isocratically eluted with 2% (v/v) isopropanol in MilliQ water at a flow rate of 1.65 ml/min. The effluent was monitored by refractive index (RI) detection. SEC-RI profiles were recorded using OpenLab CDS software. Individual retention-time intervals of collected defined HMO fractions were as follows: total HMOs, 700–1430 min; total acidic HMOs, 700–905 min; and neutral HMOs (DP6 to DP9), 1004–1154 min.

These well-defined HMO fractions were concentrated by a vacuum concentrator (ScanSpeed) and freeze-dried (Christ Epsilon 2-4LSCplus) prior to analysis.

### Instrumentation

The defined HMO fractions obtained by preparative SEC were further sub-fractionated by liquid chromatography (LC) using either hydrophilic interaction chromatography (HILIC) or porous graphitized carbon (PGC) as stationary phases.

HILIC was performed using an Acquity Premier UPLC system (Waters Corp) employing a Glycan BEH amide column (130 Å, 1.7 µm, VanGuard FIT 2.1 × 150 mm) at a solvent flow rate of 400 µl/min. The sample was eluted from the column using a binary solvent system composed of 50 mM ammonium formate at pH = 4.4 (solvent A) and acetonitrile (solvent B) at a temperature of 65 °C.

Separation using porous graphitized carbon (PGC) employed a Hypercarb™ column (150 × 0.5 mm, 5 μm, Thermo Scientific) at a solvent flow rate of 600 μl/min. The sample was eluted from the column using a binary solvent system composed of 0.1% formic acid in water (solvent A) and 0.1% formic acid in acetonitrile (solvent B) at a temperature of 70 °C.

Samples sub-fractionated by LC were directly infused via offline nano-electrospray ionization (nESI) operating in positive ion mode into a custom-built instrument that includes a cryogenic ion trap for infrared (IR) messenger-tagging spectroscopy connected to a time-of-flight (TOF) mass spectrometer (TOFWERK). After introduction, ions are directed through a series of ion funnels, accumulated and bunched into packets of ~1 ms duration, and then guided through multiple stages of differential pumping until they reach a cryogenic trap maintained at a temperature of 45 K. The trapped ions are cooled by collisions with a mixture of helium and nitrogen in an 80:20 ratio, forming weakly bound clusters with N_2_. A continuous-wave mid-IR laser (IPG Photonics) irradiates the N_2_-tagged ions for a duration of 50 ms, at which point they are released and analyzed using the TOF mass spectrometer. Upon absorption of an IR photon, energy is redistributed among the vibrational modes of the ions, leading to the dissociation of the nitrogen tag(s). We obtain an IR fingerprint spectrum of the tagged species by measuring the ratio of the tagged and untagged ion signal at each laser wavenumber step [21, 28]. We can also measure IR fingerprint spectra of the charged fragments of the precursor molecular ions produced by collision-induced dissociation (CID) in one of the ion guides before the cryogenic trap [19, 20, 23].

### HMO IR fingerprint database

To identify an HMO species in a sample, we measure its cryogenic IR spectrum and compare it with those stored in our database, using the Pearson correlation coefficient (PCC) to determine the best match. The PCC method measures the linear relationship between two vector variables. A coefficient close to 1 indicates a strong positive correlation, while a value close to 0 implies no correlation whatsoever. By calculating the PCC for our measured IR spectrum with each of our reference spectra, we determine the degree of similarity between them, facilitating the identification of the best match. Because isomeric HMOs share the same functional groups (i.e., OH- and NH- groups), unless one has infinite spectral resolution there will always be some degree of correlation between the spectra of two isomers, and this is particularly true for larger species in which the cryogenic IR spectrum is more congested. In these cases, we subtract the same broad background absorption from both the sample and reference spectra before calculating the PCC value, as this emphasizes the differences in the sharp features.

Standard reference spectra of lacto-N-fucopentaose (LNFP) I, II, and V have been previously measured [19, 29]. For this work, we measured IR spectra of three additional standards: lacto-N-hexaose (LNH), monofucosyllacto-N-hexaose (MFLNH) I, difucosyllacto-N-hexaose (DFLNH) b and fragments of the latter. All our HMO spectra (i.e., sample and reference) have been measured for sodiated species in which a sodium ion carries the charge, as these seem to be particularly abundant in mass spectra of HMOs. While in protonated oligosaccharides it has been shown that fucose can migrate in the gas phase, this does not occur in the sodiated species [30].

In cases where we do not find a match between the measured IR spectrum and one from our database because of lack of standards, we identify the precursor molecular ion by measuring IR spectra of structurally determinant fragment ions [19, 20, 23].

## Results

**Fig. 1:**
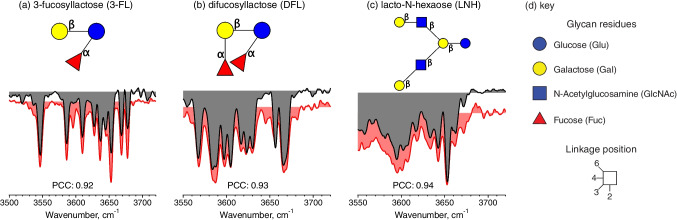
Cryogenic infrared spectra of three known HMOs found in our milk sample (gray) compared with the best-match spectra stored in our database (red) along with their respective PCC values (**a**) 3-fucosyllactose (3-FL); (**b**) difucosyllactose (DFL); and (**c**) lacto-N-hexaose (LNH); (**d**) Key for the graphical representation of glycans (SNFG)

We begin by demonstrating the fidelity of our spectroscopic fingerprinting approach by comparing IR spectra of three known glycan isomers from a pooled human milk sample with those in our database. Figure 1 shows such a comparison for 3-fucosyllactose (3-FL) (*m/z* 511), difucosyllactose (DFL) (*m/z* 657) and lacto-N-hexaose (LNH) (*m/z* 1095), with the sample spectra shown in red and the database reference spectra in gray. By eye, one can see that the match is quite compelling, and this is confirmed by the high PCC value in each case. For comparison, we show in Table S1 the PCC values obtained if one purposely mismatches a measured spectrum of a known oligosaccharide isomer with a database entry from a different isomer. While there is no formal threshold distinguishing a match from a mismatch, the comparison of the PCC values from intentionally mismatched spectra provides an indication of the range over which an assignment can be confidently determined when the relevant isomer spectrum is in our database.

For HMO isomers that are not already in our database, we use a fragmentation-based method for assigning the isomeric form and then expand the database by adding its cryogenic IR spectrum [19, 20, 23].

**Fig. 2:**
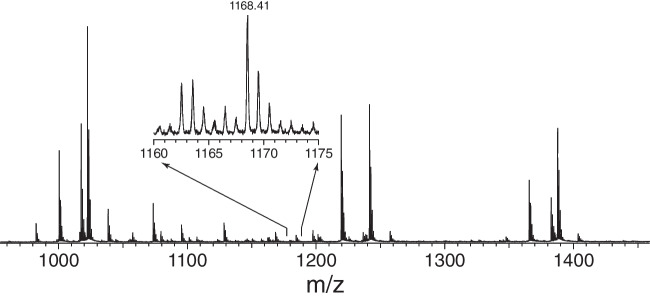
Mass spectrum of the neutral fraction of an HMO sample containing DP6-9 directly infused by nESI. The inset shows the region of *m/z* 1168, which corresponds to a sodiated oligosaccharide with 3 hexose, 1 HexNAc, and 3 fucose units

In recent all ion fragmentation LC-ESI-IM-qTOF-MS analyses of HMOs, five trifucosylated oligosaccharides were tentatively identified in a pooled human milk derived total HMO fraction, three of which are based on a lacto-N-tetraose (LNT) backbone and two based on lacto-N-neotetraose (LNnT). Because some of those structures were not consistent with the commonly known specificities of the relevant enzymes, our focus in this report was to identify all major isomers we observe at *m/z* 1168. This *m/z* corresponds to an oligosaccharide with 3 hexose, 1 N-acetylhexosamine, and 3 fucose units (i.e., the mass of lacto-N(neo)trifucoheptaoses or LN(n)TFHs). Figure 2 shows a nanoESI mass spectrum of the neutral fraction of an HMO sample in the region containing oligosaccharides with between 6 and 9 monosaccharides (i.e., DP6-9).

**Fig. 3:**
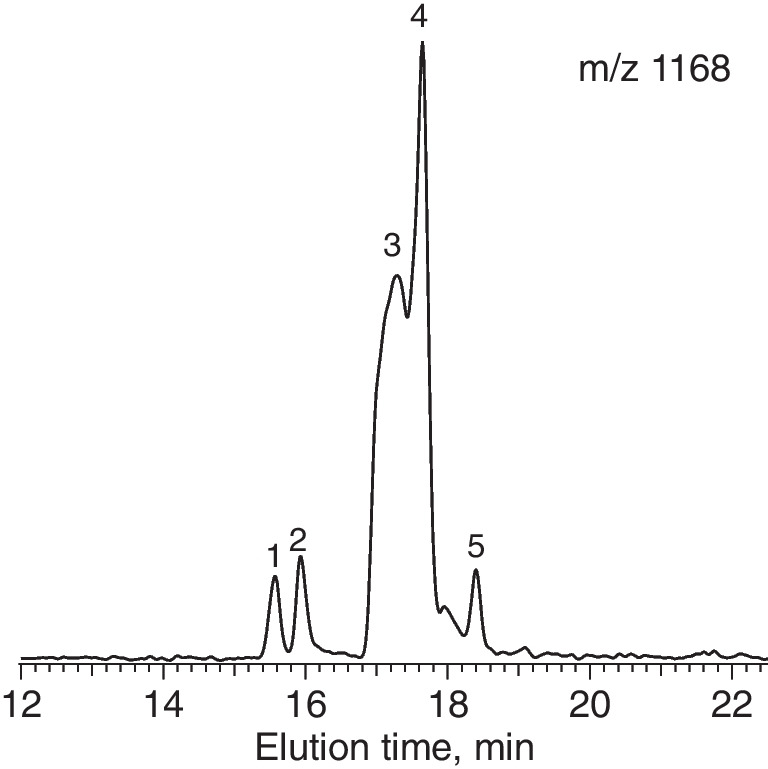
Liquid chromatogram on a PGC column illustrating multiple isomers of the oligosaccharide with *m/z* 1168

To prepare a pure sample to be analyzed by cryogenic infrared spectroscopy, we used a two-step cleaning procedure in which we first employ HILIC to separate our glycans of interest from all other glycans and then PGC to separate the isomers of the molecule with *m/z* 1168 and collect the fractions containing them. The PGC chromatogram, shown in Fig. 3, reveals at least 5 different isomers.

**Fig. 4:**
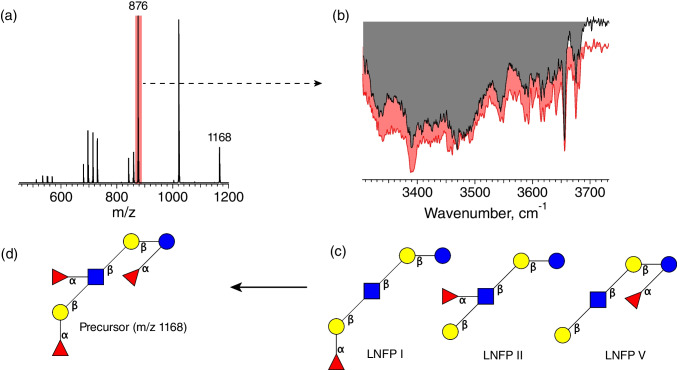
Characterization of the isomer contained in fraction 4: (**a**) Fragment mass spectrum of the *m/z* 1168 precursor ion; (**b**) cryogenic infrared spectrum of the *m/z* 876 fragment ion (gray) compared to a linear combination of database spectra (red); (**c**) LNFP isomers that contribute to the synthetic IR spectrum of (**b**) with contributions of 27% LNFP I, 50% LNFP I, 23% LNFP V; (**d**) reconstructed precursor ion structure given the three observed LNFP isomers

We began our analysis by performing CID on the fraction containing the major LC peak (labeled 4 in Fig. 3) to generate diagnostic fragment ions, the mass spectrum of which is displayed in Fig. 4(a). We then measured the cryogenic IR spectrum of the glycan fragment ions with *m/z* 876, shown in red in Fig. 4(b), which results from the loss of two fucose units from the precursor ion. Using our IR database, we found no acceptable match of this spectrum to that of any single HMO isomer with *m/z* 876. However, we could create a synthetic spectrum consisting of a linear combination of HMO isomer spectra from our database, shown in gray in Fig. 4(b), where the isomers LNFP I, LNFP II, and LNFP V (shown in Fig. 4(c)) are the contributors. The PCC value of 0.94 attests to the fidelity of this spectrum in reproducing the original. To demonstrate the uniqueness of this determination, we show in Figure S1 a different combination of LNFP isomer spectra along with its respective PCC value. Together with visual inspection, the difference in PCC reinforces our conclusion that the *m/z* 876 charged fragments of the precursor ion represent only residues corresponding to LNFP I, LNFP II, and LNFP V.

Putting this information together, we can assign the final structure of the precursor (*m/z* 1168) as Fuc(a1-2)Gal(b1-3)[Fuc(a1-4)]GlcNAc(b1-3)Gal(b1-4)[Fuc(a1-3)]Glc, shown in Fig. 4(d).

Having identified the isomeric structure of fraction 4, we then proceeded to investigate fraction 3. We found that the IR spectrum of the precursor ion has an exact match with that of the isomer of fraction 4 (see Fig. S2(c) and (d)), suggesting that these fractions contain the same species. This can be explained by the separation of the reducing-end anomers on the PGC column, as has been previously observed. In solution, the α and β reducing-end anomers of glycans are in equilibrium with one another, as the hexose ring of the terminal sugar can open and reclose, allowing interconversion of the anomers. Under the conditions of our measurements, the timescale for this mutarotation reaction in solution is on the order of hours, which is significantly longer than the separation time on the PGC column, allowing separate peaks to elute for the different anomers. However, the process of collecting fractions and analyzing them gives time for anomeric re-equilibration. This means that the infrared spectra that we measure will always be a mixture of the α and β reducing-end anomers [22].

**Fig. 5:**
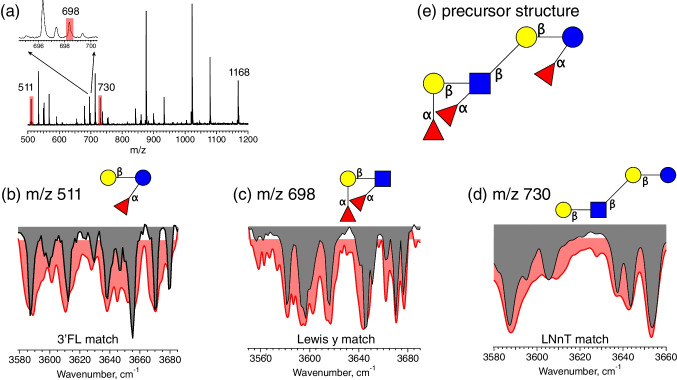
Characterization of fraction 5. (**a**) Fragment mass spectrum of the *m/z* 1168 precursor ion; (**b**) IR spectrum of the *m/z* 511 fragment ion (gray) compared to the best fit database spectrum (red); (**c**) IR spectrum of the *m/z* 698 fragment ion (gray) compared to the best fit database spectrum (red); (**d**) IR spectrum of the *m/z* 730 fragment ion (gray) compared to the best fit database spectrum (red); (**e**) reconstructed precursor structure given the three observed fragment isomers

Moving on to fraction 5, we apply the same identification procedure—first fragmenting the *m/z* 1168 precursor ion (Fig. 5(a)) and then measuring infrared spectra of the fragment ions. However, no definitive match from fragments at *m/z* 876 was obtained nor could we generate an acceptable match from a linear combination of spectra from our database. This demonstrates that not all isomers with *m/z* 876 are contained in our database. For this reason, we examined other fragments to provide information on the precursor isomeric structure. Looking at the *m/z* 730 fragment ion, we find a match with the oligosaccharide LNnT (Fig. 5d), which provides the backbone structure. We then determine the fucose locations from the fragments with *m/z* 511 and *m/z* 698 (Fig. 5(b) and (c), respectively). The IR spectrum of the *m/z* 511 fragment ion matches that of 3-FL (Gal(b1-4)[Fuc(a1-3)]Glc), whereas that of *m/z* 698 corresponds to the spectrum of Lewis y tetrasaccharide (Fuc(a1-2)Gal(b1-4)[Fuc(a1-3)]GlcNAc). Combining the identified fragment structures and assuming no fucose migration, we deduce the precursor molecule to be Fuc(a1-2)Gal(b1-4)[Fuc(a1-3)]GlcNAc(b1-3)Gal(b1-4)[Fuc(a1-3)]Glc, shown in Fig. 5e.

**Fig. 6:**
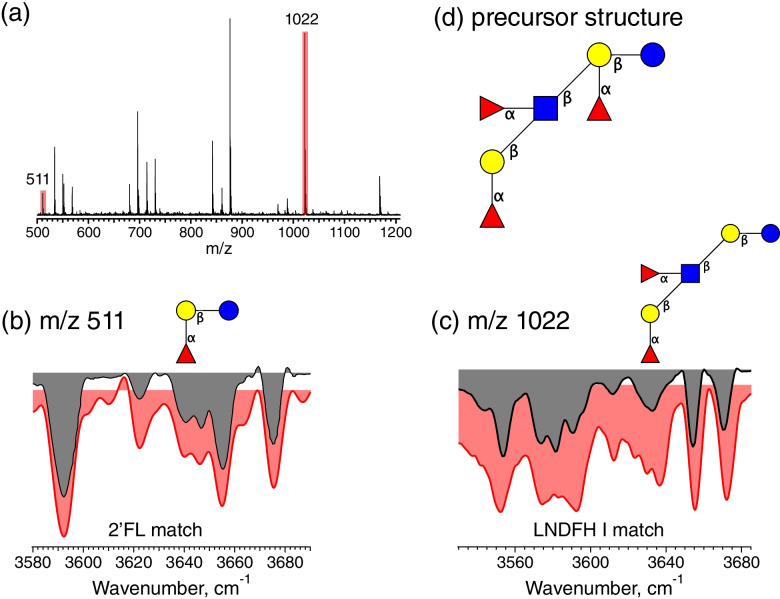
Characterization of fraction 1. (**a**) Fragment mass spectrum of the *m/z* 1168 precursor ion; (**b**) IR spectrum of the *m/z* 511 fragment ion (gray) compared to the best fit database spectrum (red); (**c**) IR spectrum of the *m/z* 1022 fragment ion (gray) compared to the best fit database spectrum (red); (**d**) reconstructed precursor structure given the three observed fragment isomers

For fraction 1, CID analysis also reveals diagnostic fragments that aid in its structural identification. The IR spectra of the *m/z* 511 and 1022 fragment ions (Fig. 6(b) and (c)), which must have been produced by parallel dissociation pathways, match those of 2′-FL and LNDFH I from our database, respectively. LNDFH I has an LNT backbone that includes two fucose units: one attached to the GlcNAc via an α1-4 linkage and the other to the terminal Gal via α1-2 linkage. 2′-FL has a fucose unit attached at the terminal Gal (Fuc(a1-2)Gal(b1-4)Glc). Taking the information from these two fragment ions together allows us to identify the precursor structure as Fuc(a1-2)Gal(b1-3)[Fuc(a1-4)]GlcNAc(b1-3)[Fuc(a1-2)]Gal(b1-4)Glc, which is shown in Fig. 6(d).

**Fig. 7:**
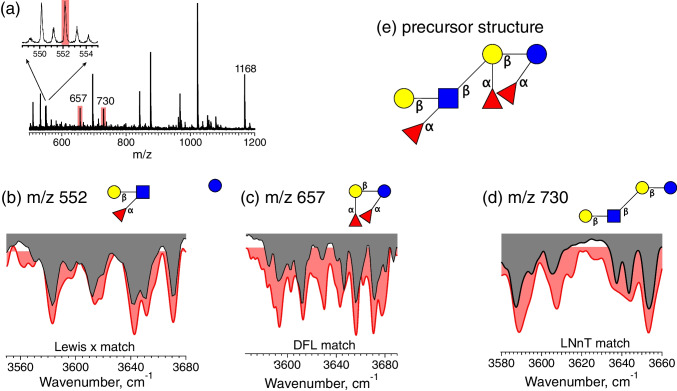
Characterization of fraction 2. (**a**) Fragment mass spectrum of the *m/z* 1168 precursor ion; (**b**) IR spectrum of the *m/z* 552 fragment ion (gray) compared to the best fit database spectrum (red); (**c**) IR spectrum of the *m/z* 657 fragment ion (gray) compared to the best fit database spectrum (red); (**d**) IR spectrum of the *m/z* 730 fragment ion (gray) compared to the best fit database spectrum (red); (**e**) reconstructed precursor structure given the three observed fragment isomers

Finally, for fraction 2, the IR spectrum of the *m/z* 730 fragment ion (Fig. 7(d)) identifies the backbone as LNnT; that of the *m/z* 657 fragment ion (Fig. 7(c)) indicates the presence of DFL, and the spectrum of the fragment ion with *m/z* 552 matched that of Lewis x trisaccharide. Together, these fragments indicate that fraction 2 corresponds to the HMO isomer Gal(b1-4)[Fuc(a1-3)]GlcNAc(b1-3)[Fuc(a1-2)]Gal(b1-4)[Fuc(a1-3)]Glc, as shown in Fig. 7(e).

It is important to note that in all cases our analysis assumes that fucose migration does not occur in the sodiated charge state, which is consistent with previous observations. This assumption is clearly validated by the fact that apart from the reducing-end anomers in fractions 3 and 4, all other isomers of the HMO at *m/z* 1168 exhibit distinct cryogenic IR spectra (Figure S2). If fucose residues were migrating, this would cause interconversion of the HMO isomers that would reveal itself as a mixture of IR spectra, which is not the case.

## Discussion and conclusions

Three of the four LN(n)TFH isomers that we observe with *m/z* 1168 (in their sodiated form) (fractions 4, 1, and 2, shown in Figs. 4, 6, and 7 respectively) have been tentatively identified in human milk by 4D LC-ESI-IM-qTOF-MS [31]. Their assignment was not definitive because they did not yield all possible cross-ring fragment ions to determine all the linkages, some of which represent exceptions to the known specificities of the relevant enzymes [31]. Moreover, their LC-CID MS/MS approach is unable to distinguish epimers that may be present. The use of cryogenic infrared spectroscopy has allowed us to determine definitively the assignment of these structures (Table S2), since at low temperatures the pattern of spectral features is unique to a given isomer/epimer [19, 20, 23]. In this respect, it is noteworthy to mention that this approach works without the need of standards for the precursor molecule, which may not be available. Precursor ions are fragmented to smaller species, the spectra of which are compared to those of standards in our database. Reconstruction of the precursor from such structurally determinant fragment ions unambiguously determines the isomeric structure of the former. While this combination of techniques requires additional measurement time, the fragmentation procedure only has to be performed once for each newly identified molecule, since we add the IR spectrum of the precursor to our database. Subsequent detection of these species can be done by a simple database search.

The fourth structure that we identify (fraction 5, Fig. 5e) is not reported in the work of Gonsalves et al. [31], perhaps because of its low abundance. Moreover, we do not observe two of the structures they report, labeled as X1 and X2 in their paper [31].

Two of the LN(n)TFH isomers that we have identified, Fuc(a1-2)Gal(b1-3)[Fuc(a1-4)]GlcNAc(b1-3)[Fuc(a1-2)]Gal(b1-4)Glc (Fig. 6(d)) and Gal(b1-4)[Fuc(a1-3)]GlcNAc(b1-3)[Fuc(a1-2)]Gal(b1-4)[Fuc(a1-3)]Glc (Fig. 7(e)) do not fit accepted anabolic pathways for HMOs with respect to known substrate specificities of the relevant enzymes. The β−1,3-N-acetylglucosaminyltransferase family of enzymes (*B3GNTs*, EC 2.4.1.149) extends the lactose core by catalyzing the addition of a GlcNAc unit to the terminal galactose residue via a β−1,3 linkage. They recognize a specific configuration of the acceptor molecule, which includes a free galactose. Prior fucosylation of the terminal galactose alters its structure and thus should prevent proper interaction with the enzyme’s active site, likely by steric hindrance. Moreover, the *FUT2* encoded fucosyltransferase (EC 2.4.169), which adds fucose via an α−1,2 linkage, also requires a substrate with a terminal galactose. According to the substrate specificities of B3GNTs and FUT2, the HMOs shown in Figs. 6(d) and 7(e) should not exist. Our findings confirm the existence of at least two isomers with an α1-2 fucosylated Gal penultimate to the reducing end of these HMOs, as suggested by Gonsalves et al. based on their 4D LC-ESI-IM-qTOF-MS analyses [31]. We hypothesize that either the substrate specificities of the B3GNTs and FUT2 encoded glycosyltransferases may not be as strict as assumed until now or that alternative enzymatic pathways may exist that could explain the synthesis of these unusual HMOs with α 1-2 fucosylated Gal penultimate to the glycans’ reducing end.

These results demonstrate the importance of an isomer-specific detection method such as ours that may enable a more comprehensive understanding of the biosynthetic pathways of human milk oligosaccharides. Moreover, complete structural elucidation of novel HMO structures by the approach we have developed might help to inspire subsequent preclinical or clinical studies of these novel glycan structures. This in turn could be key to revealing possible new benefits that these biomolecules may contribute to healthy early-life development and innovations in the field of nutrition.

## Supplementary Information

Below is the link to the electronic supplementary material.

ESM 1 (PDF 3.56 MB)

## Data Availability

The data is available from the corresponding author at thomas.rizzo@epfl.ch.

## Declarations

### Source of biological samples

Defined HMOs fraction have been isolated from anonymized pooled human milk samples as described in the “Methods” section and elsewhere (Mank, M., P. Welsch, A. J. R. Heck and B. Stahl (2019). “Label-free targeted LC-ESI-MS(2) analysis of human milk oligosaccharides (HMOS) and related human milk groups with enhanced structural selectivity.” *Anal Bioanal Chem* 411(1): 231–250).

The usage and analysis of defined HMO fractions were performed in accordance with ethical standards and guidelines as laid down in the Declaration of Helsinki. Ethical approval and written consents of donors were given as stated in the publications by Thurl et al. (see Thurl S, Henker J, Siegel M, Tovar K, Sawatzki G. Detection of four human milk groups with respect to Lewis blood group dependent oligosaccharides. *Glycoconjugate J.* 1997;14(7):795–9.; Thurl S, Munzert M, Henker J, BoehmG,Muller-Werner B, Jelinek J, et al. Variation of human milk oligosaccharides in relation to milk groups and lactational periods. *Br J Nutr.* 2010;104(9):1261–71. 10.1017/S0007114510002072.; Thurl S, Henker J, Taut H, Tovar K, Sawatzki G. Variations of neutral oligosaccharides and lactose in human milk during the feeding. *Z Ernahrungswiss*. 1993;32(4):262–9).

The same samples were already subjected to HMO analysis in these prior studies. They were now used for the same purpose for which ethical approval was received as already stated in the former publications by Thurl et al. (see Thurl S, Munzert M, Henker J, BoehmG,Muller-Werner B, Jelinek J, et al. Variation of human milk oligosaccharides in relation to milk groups and lactational periods. *Br J Nutr.* 2010;104(9):1261–71. 10.1017/S0007114510002072.; Thurl S, Henker J, Taut H, Tovar K, Sawatzki G. Variations of neutral oligosaccharides and lactose in human milk during the feeding. *Z Ernahrungswiss*. 1993;32(4):262–9).
