# Supplementary material for: Characterization of rare trifucosylated human milk oligosaccharides by cryogenic infrared ion spectroscopy (CIRIS)
Source: Anal Bioanal Chem. 2025 Sep 26;417(27):6231–9. doi: 10.1007/s00216-025-06114-5 (PMC12583426; doi:10.1007/s00216-025-06114-5)
Supplement: Supplementary file 1 — (PDF 3.56 MB) [file 216_2025_6114_MOESM1_ESM.pdf]

Supplementary Information

Characterization of rare trifucosylated human milk oligosaccharides by cryogenic infrared ion spectroscopy (CIRIS)

Ali H. Abikhodr[1], Stephan Warnke[1], Ahmed Ben Faleh[1], Thomas R. Rizzo[1,2], Sibel Goeraler[3], John Gonsalves[3], Bernd Stahl[3, 4], Marko Mank[3]

[1]Isospec Analytics SA, CH-1020 Renens, Switzerland

[2]École Polytechnique Fédérale de Lausanne, EPFL SB ISIC, Station 6, CH-1015 Lausanne, Switzerland

[3]Danone Research and Innovation, 3584 CT Utrecht, The Netherlands

[4]Department of Chemical Biology and Drug Discovery, Utrecht Institute for Pharmaceutical Sciences, Utrecht University, 3584 CG Utrecht, The Netherlands

Table S1 below shows the PCC values for intentionally mis-matched oligosaccharide spectra. While there is formally no threshold defined for what constitutes a match, cases in which PCC values are below 0.7 should be examined visually. It should be noted that for larger HMOs, broad background absorption at lower wavenumber can artificially push the PCC value higher, partially masking the differences in sharp structure. In these cases, performing a baseline correction before determining the PCC allows for a more critical comparison of spectra, lowering the PCC in mismatched cases.

Table S1 – PCC values for intentionally mismatched spectra

**HMO pairs**	**PCC value**
2'-FL and 3-FL	0.09
LNFP I and LNFP II:	0.37
LNFP III and LNFP V	0.30
DFLNnH and DFLNHc	0.30
DFLNHa and DFLNHb	0.16

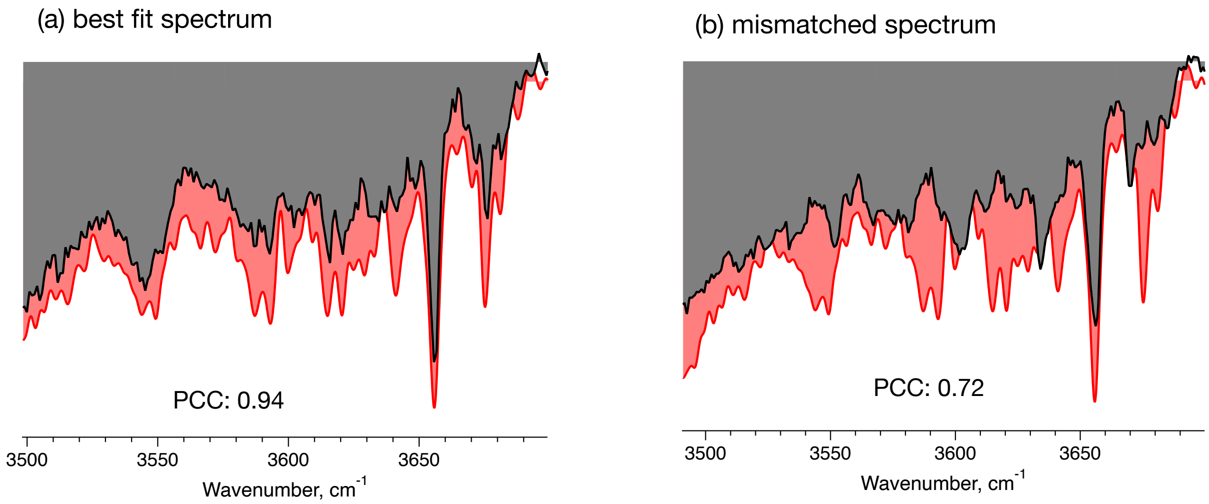

Figure S1. (a) Measured cryogenic IR spectrum of the *m/z* 876 fragment from fraction 4 (red) compared with the best fit combination from all database spectra (gray) with contributions of 27% LNFP I, 50% LNFP II, 23% LNFP V; (b) Measured cryogenic IR spectrum of the m/z 876 fragment of fraction 4 (red) compared with a fit that includes LNFP I,

LNFP III, and LNFP VI.

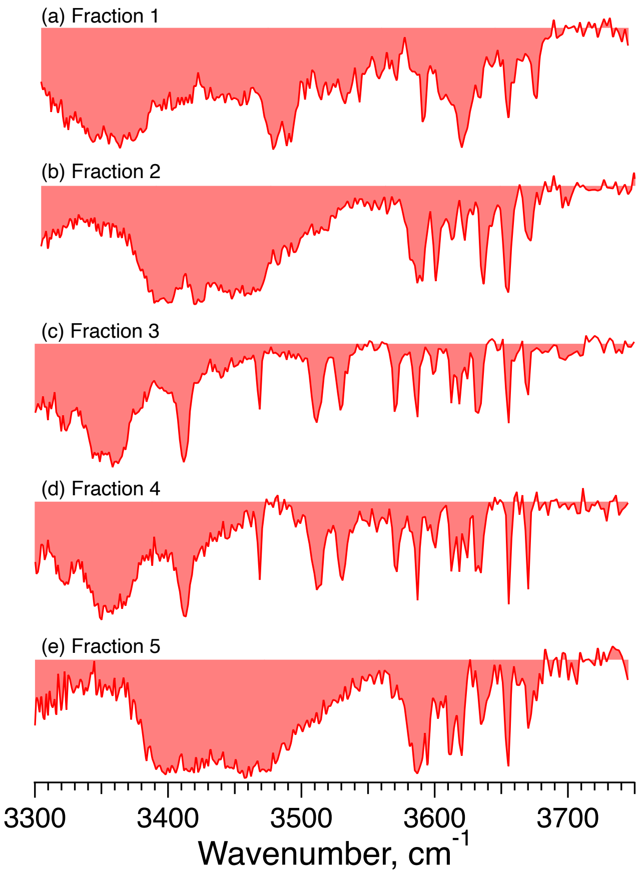

Figure S2. (a)-(e) Measured cryogenic IR spectrum of the precursor molecules of the 5 different fractions (i.e., isomers) with *m/z* 1168, respectively. As explained in the text, fractions 3 and 4 represent a mixture of the two reducing end anomers of Fuc(a1-2)Gal(b1-3)[Fuc(a1-4)]GlcNAc(b1-3)Gal(b1-4)[Fuc(a1-3)]Glc. The fact that all the others are different indicates that fucose migration does not occur in the sodiated species

Table S2 below compares the LN(n)TFH isomers that we observe with those tentatively identified by Gonsalves et al. (Ref 27).

Table S2

This work	Gonsalves, J.; Bauzá-Martinez, J.; Stahl, B.; Dingess, K. A.; Mank, M. *Analytical Chemistry* **2025**, *97*, 5343-5836.
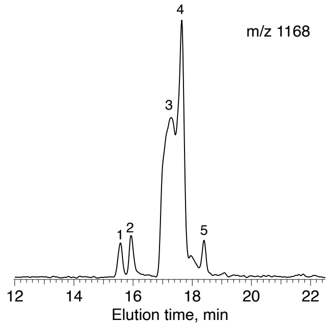	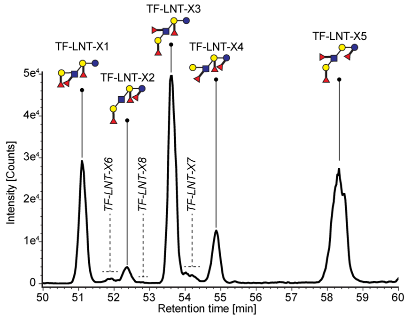
Figure 4 (fraction 4) 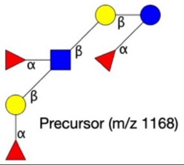	Structure X5 (LNT backbone) 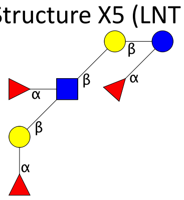
Figure 5 (fraction 5) 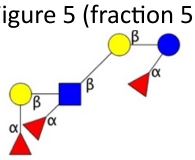	**No corresponding structure**
Figure 6 (fraction 1) 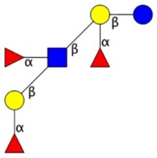	Structure X3 (LNT backbone) 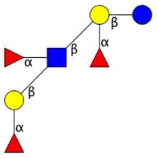
Figure 7 (fraction 2) 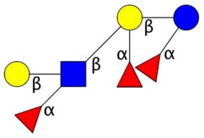	Structure X4 (LNnT backbone) 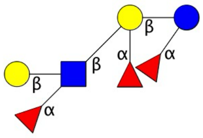
	Structure X1 (LNnT backbone) 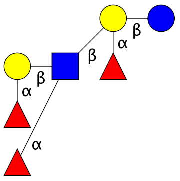 [1]Lacto-N-neotrifucoheptaose II
	Structure X2 (LNT backbone) 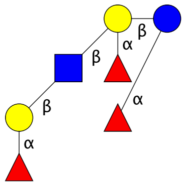

[1]Hallgren, P. and A. Lundblad (1977). "Structural analysis of oligosaccharides isolated from the urine of a blood group A, secretor, woman during pregnancy and lactation." Journal of Biological Chemistry 252(3): 1023-1033.
